# Measurement of prospective memory in Spanish speakers

**DOI:** 10.3389/fnhum.2023.1126039

**Published:** 2023-05-02

**Authors:** Laura Cadavid, Alicia Camuy, Valerie Velez, Sarah Raskin

**Affiliations:** ^1^Department of Psychiatry, Boston Children’s Hospital, Boston, MA, United States; ^2^Icahn School of Medicine at Mount Sinai Medical Center, Department of Neurology, New York, NY, United States; ^3^Department of Neurology, Columbia University, New York, NY, United States; ^4^Department of Psychology and Neuroscience Program, Trinity College, Hartford, CT, United States

**Keywords:** prospective memory (PM), episodic future thinking, acculturation, neuropsychology, Spanish sample

## Abstract

**Introduction:**

This study aimed to provide preliminary evidence on the psychometric properties of a measure of prospective memory in Spanish speakers, the Memory for Intentions Test (MIST) Spanish translation.

**Methods:**

In addition, this study investigated whether acculturation influenced performance on the MIST. Finally, we measured other cognitive factors that might be impacting the relationship between culture and prospective memory performance. These factors were working memory, autobiographical memory, and episodic future thought.

**Results:**

Overall, the psychometric properties of the Spanish MIST appear to be similar to the English language MIST, but our sample size was too small to allow for the creation of a normative database. The MIST recognition item was significantly related to years of education and years of speaking either Spanish or English.

**Discussion:**

This suggests a need to investigate ways to improve the test to eliminate these effects. In addition, acculturation was related to the measure of episodic future thought.

## Introduction

The influence of culture has been demonstrated on a variety of aspects of memory functioning (e.g., Nisbett and Masuda, 2003; Ostrosky-Solís et al., 2004; Alea et al., 2019). The effects of culture go beyond language differences and many authors have pointed out the need for culturally appropriate neuropsychological tests to be more than simple language translations (e.g., Alea and Wang, 2015). This has been demonstrated also with tests that were once argued to be culture free (Rosselli and Ardila, 2003; Arentoft et al., 2012).

In a review of studies of neuropsychological measures in Spanish speakers, Gasquoine (2001) suggests that test differences that have been observed are due to educational differences, non-equivalent translations, and acculturation, and that these factors should be taken into account when providing Spanish versions of tests developed in English. In addition to culturally sensitive test construction, researchers have suggested the need to obtain adequate normative data from native speakers and from those living in countries in Latin America (e.g., Rivera et al., 2019).

This is especially important, as recent studies have demonstrated that minorities are more likely to be given a false-positive diagnosis of cognitive impairment than non-Hispanic white participants due to artificially low neuropsychological test scores (Boone et al., 2007; Arentoft et al., 2012). This reinforces the need for local norms and measurement of multivariate base rates of low scores (Rivera et al., 2019). One factor is acculturation (Arentoft et al., 2012). Acculturation is defined as the process of both psychological and behavioral changes that occur after an individual has had prolonged contact with a new culture (Zea et al., 2003). Previous studies have found acculturation into United States (U.S.) (dominant) culture is correlated with better neuropsychological performance on a variety of neuropsychological domains, including information processing (Razani et al., 2007), processing speed and verbal fluency (Arentoft et al., 2012), and working memory (Coffey et al., 2005). In contrast, higher Hispanic/Latino acculturation has been associated with better performance on measures of memory (Arentoft et al., 2012). Therefore, it is important to recognize any effects that acculturation may have on performance as findings can help assess, treat and diagnose this population. The need to determine the influence of social determinants like education and acculturation is particularly critical as Hispanic/Latino brain injury survivors have been found to have worse functional outcomes (Arango-Lasprilla et al., 2007) and to be less likely to receive treatment and be employed than white survivors (Gary et al., 2009).

When assessing minority groups (Hispanic, Asian, and Middle-Eastern descent), one study reported that monolingual English-speaking Anglo-Americans consistently outperformed other ethnic groups (Razani et al., 2007). More importantly, this study found a correlation between performance on common measures of information processing and attention and higher acculturation variables, such as time educated in the United States (U.S.) and amount of English spoken when growing up. These findings indicate effects of acculturation on cognition related to both language and non-linguistic cultural factors. Similar findings were observed in Hispanic/Latino individuals specifically. When assessing effects of acculturation in HIV + Latino individuals, Arentoft et al. (2012) found that higher U.S. acculturation scores were associated with better overall neuropsychological, verbal fluency, processing speed and attention. However, this same study also found higher Latina/o acculturation was correlated with better memory performance (Arentoft et al., 2012).

Toward this end we wanted to investigate the psychometric properties of the Spanish translation of the Memory for Intentions Test (MIST) and determine any effects of preferred language, education, and acculturation on this measure. In addition, to better understand the influence of culture on prospective memory, we measured related cognitive functions that we judged priori to be possible mediators of any relationship between culture and prospective memory. These were working memory, autobiographical memory, and episodic future thought.

There are very few standardized clinical tests of PM available and of those, to our knowledge, the only one available in Spanish is the Memory for Intentions Test (MIST) (Raskin et al., 2010). There is a Spanish language PM test, El Condor (Taussik, 2002), that has shown sensitivity to multiple sclerosis (Cores et al., 2017), but we do not know of normative data or if it is available for clinical use.

In a previous study of culture and PM, we administered the MIST to four groups of participants all living in the U.S.: a healthy group that had immigrated from the Caribbean; a healthy group whose parents had immigrated from the Caribbean; a healthy group of European descent; and a group of European descent who had been diagnosed with traumatic brain injury (TBI) (Alea et al., 2019). Differences were found such that those born in the Caribbean were superior on the 24-h recall task but significantly worse on the time-based items. We speculated that there might be cultural contributions to differences in time-perception. Using the English language MIST, Tureson et al. (2021) found significant relationships between acculturation and performance when comparing Hispanic/Latino and non-Hispanic/Latino participants living with HIV.

Extensive data on the psychometric properties of the English-language MIST are published in the manual (Raskin et al., 2010) as well as additional data by Woods et al. (2008) and Raskin (2009). Coefficient alpha for the eight MIST Prospective Memory Trials was 0 71 for the standardization sample. All intercorrelations of the eight trials were significant at the *p* < 0.01 level and reflect consistent item content. Raskin (2009) reported that The MIST was validated against the two items of the Rivermead Behavioral Memory Scale that involve PM (Wilson et al., 1985). The correlation of the MIST with the two items of the Rivermead was 0.80. The test–retest reliability of the MIST was demonstrated in a group of 20 subjects who were given the MIST on two occasions 2 weeks apart. Inter-form reliability, comparing Form A and Form B in the same group of 20 subjects, was found to be 0.89.

The MIST has also been shown to have good construct validity in both younger and older adults (Kamat et al., 2014). The MIST has demonstrated significant correlations with measures of instrumental activities of daily living (e.g., Woods et al., 2008), measures of functional skills (e.g., Twamley et al., 2008), medication adherence (e.g., Raskin et al., 2014); appointment attendance (Gromisch et al., 2023), and diary studies of PM in daily life (Raskin and Sohlberg, 2009; Raskin et al., 2012).

To our knowledge, despite several language translations of the MIST, only two so far have published psychometric information. Similar to the English-language version, the Czech translation of the MIST was reported to have a summary score correlated at a medium level with neuropsychological measures including memory retention, mental flexibility, and resistance to interference (all rho = 0.37–0.42; all *p* < 0.05) (Bezdicek et al., 2014). The reliability of MIST in terms of internal consistency was insufficient when analyzing the eight individual MIST trials (α = 0.50), as was split- half reliability (split- half reliability = 0.56). In contrast, there was a high degree of reliability between six subscales classified by type (delay, cue, and mode of response; α = 0.88, split- half = 0.95). The Portuguese translation was reported to have reliability of PM scores of 0.43 in the healthy adult group, and there was a within-subject effect of task, *F*(7,553) = 27.6, *p* < 0.001, η*p*^2^ = 0.26 (Belmar et al., 2020). *Post-hoc* tests with Bonferroni correction showed items 1, 2, 3, 5, 6, and 7 had higher scores than items 4 and 8. A publication with the Italian translation does not report on psychometric properties but does mention that there were no correlations between MIST scores and education or other demographic variables (Palermo et al., 2020). Given this, it is important to look at the psychometric properties of the Spanish MIST, in addition to any significant effects of acculturation.

In addition, we used the Comprehensive Assessment of Prospective Memory (CAPM), translated into Spanish, for this study to measure self-reported PM ability. To measure cognitive functions presumed to underlie successful PM performance we also measured working memory, autobiographical memory, and episodic future thought. Working memory, also, has demonstrated effects of language and culture (e.g., Ponton et al., 2000; Puente and Ardila, 2000; Ostrosky-Solís and Lozano, 2006; López et al., 2016). Autobiographical memory, consisting of reconstructed mental representations (Schrauf, 2000), has also been demonstrated to be affected by language and culture (e.g., Bluck and Alea, 2011; Alea and Wang, 2015; Esposito and Baker-Ward, 2016). Episodic future thought is the ability to visualize future events that may occur in one’s life (Atance and O’Neill, 2001; Szpunar, 2010). We demonstrated in Alea et al. (2019) that participants born in the Caribbean were superior at describing collective future thought details, but those born in the U.S. were superior at describing episodic future thought details.

These different forms of memory reviewed above are not independent but interrelated. The constructive-episodic-simulation hypothesis suggests that it is memories of past events that are used to construct possible future events (Suddendorf and Corballis, 1997). For example, working memory has been shown to effect both autobiographical memory and future thought (Hill and Emery, 2013). These authors suggest that working memory is involved in the construction of a future event but not the actual event details.

This relationship has been shown to be influenced by culture (Wang et al., 2011). Euro-Americans were able to recall more specific details about episodic events than Chinese participants, and this was true for both past and future events (Wang et al., 2011). Cultural differences and bilingualism have been shown to impact performance on working, prospective and autobiographical memory as well as future thought. Thus, as part of our development of a Spanish language version of the MIST, we assess the effects of acculturation on these types of memories in a single study to obtain a more comprehensive understanding of the role culture plays on the types of memory performance that impact PM performance. Given the extensive Spanish speaking population in the U.S., evaluating these effects on Spanish speakers with varying degrees of acculturation to U.S. culture will help with clinical assessment as well.

Thus, the goals of the current study are two-fold. First, to provide information on the psychometric properties of the Spanish MIST. Second, to investigate the potential cognitive mediators of the relationship between culture and prospective memory. Specifically, we measured working memory, autobiographical memory, and episodic future thought. We hypothesized that: (1) The subscales of the Spanish MIST would show significant intercorrelation; (2) U.S. acculturation would be significantly positively related to performance on Digit Span, MIST, the TALE, and Future Thinking; (3) Hispanic/Latino acculturation would be significantly negatively related to Digit Span, MIST, the TALE, and episodic future thinking.

## Materials and methods

### Participants

This study was approved by the Trinity College Institutional Review Board. Participants were recruited through flyers, verbal invitation and an online Trinity College newsletter. The requirements for participating in the study were the following: participants had to be fluent Spanish speakers, over 18 years of age and younger than 80 years of age, and have no previous neurological or psychological disorder. Participants had cultural ties with Puerto Rico, Central America, and South America. Each participant was provided with written informed consent prior to being administered any study measure. Each participant received a $10 gift card as compensation for their time. Participants (*n* = 48) were of ages 18–80. Demographic and background information on the participants can be seen in Table 1.

**TABLE 1:** Participants’ demographics *N* = 50.

	Mean (s.d.) or *n*	Range
Age (years)	29.69 (17.01)	18–80
Gender (F/M/NB)	34/16/0	–
Born in U.S. (yes, no, U.S. territory)	27/18/5	–
First language (Spanish/English/Both)	32/14/4	–
Race (white, Black, Asian, mixed or other)	33, 13, 4	–
Education (years)	14.67 (3.42)	0–21
Education in U.S. (years)	10.52 (6.30)	0–21
Education in English (years)	11.03 (5.93)	0–27
Education in Spanish (years)	6.59 (6.07)	0–21
Years speaking English	18.90 (13.96)	0–68
Years speaking Spanish	26.70 (18.45)	3–79
AMAS-total U.S. acculturation	3.20 (0.56)	1.86–4.00
AMAS-total acculturation to country of origin	2.24 (0.48)	2.24–4.00
Language of thought (Spanish/English/both)	10/21/19	–
Preferred language (Spanish/English/same)	15/24/11	–
Language spoken in the home (Spanish/English/both/other)	27/27/10/2	–

AMAS, Abbreviated Multidimensional Acculturation Scale; U.S., United States; F, female; M, male; NB, non-binary.

### Materials

Participants completed a background information form that included questions on their life inside and outside of the U.S. and their exposure to both English and Spanish, such as, “Were you born in the United States? If not, how long have you lived in the United States?” and “Which language do you think in?” Participants also completed the Spanish Abbreviated Multidimensional Acculturation Scale (AMAS) (Zea et al., 2003). This included statements about the individuals’ relationship to American culture and their own “mother” culture such as “I feel as though I am a part of American culture.” Participants were provided with four possible answers ranging from “Strongly disagree” to “Strongly agree.” Assessments were also administered for each type of memory being tested: working, prospective, autobiographical memory, and future thought.

#### Prospective memory

The MIST (Raskin et al., 2010) is a 30-min, 8-trial test during which participants engage in a word search puzzle as the ongoing task. A complete description of the MIST administration and scoring procedures can be found in Raskin (2009). Briefly, it is comprised of four trials with event-based cues (e.g., “When I hand you a post-card, self-address it./Cuando le entregue una tarjeta, escríbale su dirección, como si fuera a enviársela a sí mismo/a”) and four trials with time-based cues (e.g., “In 15 min, tell me it is time to take a break./En 15 min dígame que es hora de tomar un receso”), with each item scored from 0 to 2 points; thus, the separate event-based and time-based scales have scores ranging from 0 to 8. The time- and event-based trials are balanced for delay interval (i.e., 2- and 15-min delay periods) and response modality (i.e., verbal and action responses). The MIST allows for separate scoring of time-based trials (8 points possible), event-based trials (8 points possible), 2-min delay periods (8 points possible), 15-min delay periods (8 points possible), verbal response trials (8 points possible), and action response trials (8 points possible), which are summed for a total of 48 possible points. However, this involves inclusion of the score of each trial three times in the total score (e.g., Trial 1 is a 2-min delay trial, time-based cue, and verbal response, thus contributing to the 2-min delay, time-based cue, and verbal response scores). A large digital clock is in full view of the participant at all times. For the event-based trials, the cues all have high cue-action relatedness and were considered to be ecologically relevant, meaning they are related to the response required and could naturally elicit that required response (e.g., When I hand you a request for records form, please write your doctors’ names on it). The ongoing task is non-focal as the word search is not related to the PM items. Prior studies support the reliability (Woods et al., 2008; Raskin, 2009) and construct validity (e.g., Woods et al., 2008; Raskin et al., 2010) of the MIST.

At the completion of the eight MIST trials, participants are given eight multiple choice recognition items (e.g., “At any time during this test, were you supposed to: (1) tell me to make an appointment; (2) tell me when I can call you tomorrow; (3) tell me to call for a prescription.”). The recognition scale is included as a way to determine whether PM failures are due to encoding versus retrieval failures. Impairment on recognition items is likely to reflect deficits in RM rather than PM functions. Furthermore, a 24-h delay trial was administered for which examinees were instructed to leave a voicemail message for the examiner the day after the exam indicating the number of hours the participant slept the night after the evaluation. In addition, the following error types were coded: (1) no response (i.e., response omission errors); (2) task substitutions (e.g., replacement of a verbal response with an action or vice-versa); (3) loss of content (e.g., acknowledgment that a response is required to a cue, but failure to recall the content); and (4) loss of time (i.e., performance of an intention greater than ± 15% before or after the target cue). If the participant makes no response to the PM cue, those are coded as “no response” errors and are presumed to be directly due to failure of PM (i.e., cue detection). Task substitution errors (e.g., intrusions and perseverations) are likely multi-determined, but presumed to be due to executive control deficits (e.g., Carey et al., 2006). Loss of content errors most likely reflect retrospective memory failures and loss of time errors seem to be due to difficulty with strategic monitoring or timing.

The MIST was translated into Spanish by John Wiebe at the University of El Paso and is available from Psychological Assessment Resources (PAR). He reports that he used certified independent translators to do the translation and the back translation. They then conducted a committee resolution process with both translators, a fluently bilingual psychology doctoral student (ABD), and himself, reaching consensus on all points. There are no normative data available at this time.

The CAPM is a self-report questionnaire used to assess PM (Chau et al., 2007). It takes approximately 10–15 min to complete. In this study we used only Section A containing 39 items relating to frequency of PM failure in the last month. Items are rated on a five-point scale. This scale indicates that 1 = “never,” 2 = “rarely,” 3 = “occasionally,” 4 = “often,” and 5 = “very often.”

Most items in Section A can be categorized into one of two subscales, independent activities of daily living (IADL) and basic activities of daily living (BADL), established by Waugh (1999) using a principal component analysis. There are 23 items relating to IADL, such as “Leaving the iron on” and “Not remembering to pay bills.” For the BADL subscale there are 10 items such as “Not locking the door when leaving home” and “Leaving water taps on.” Given the “not applicable” category allowed, total scores and subscales scores were not used. Instead, for each participant three scores were calculated (total CAPM, IADL subscale, and BADL subscale) by summing the participant’s ratings on the 1–5 scale for all completed items and dividing by the total number of items not including the items marked “not applicable.” Therefore, the possible range for mean total and subscale CAPM scores was 1–5, with higher scores indicating more frequently perceived PM failure. Two independent native Spanish speakers translated and back translated the CAPM into Spanish.

#### Working memory

For working memory, the Digit Span Forward (DSF) and Digit Span Backward (DSB) subtests from the Escala de Inteligencia de Wechsler para Adultos-III (Wechsler, 2008) were administered according to standard protocols. In the DSF, the researcher reads a series of numbers to the participant, the participant then repeats as many of the digits in the correct order as they can remember. The DSB follows the same protocol, however, the participant is asked to repeat the digits back to the researcher in the opposite order of their delivery. In all cases the test is discontinued after two errors of the same length number string.

#### Autobiographical memory

The TALE scale (Bluck and Alea, 2011) was designed to be a brief measure of the functions of autobiographical memory. In the TALE, the participant is asked to answer a series of questions regarding the frequency at which they recall past events for specific reasons such as “I think back over or talk about my life or certain periods of my life when I want to feel that I am the same person as before” or “I think back over or talk about my life or certain periods of my life when I want to develop more intimacy in a relationship.” Each question asks the frequency based on a specific reason and the participant is asked to choose a response between almost never, seldom, occasionally, often and very frequently. Responses are scored on three sub-scales: Self-continuity, Social-Bonding, and Directing Behavior. The TALE was translated into Spanish and then back-translated to check for errors or ambiguity by two independent native Spanish language speakers.

#### Episodic future thought

Episodic future thought (EFT) was assessed by asking the participant to imagine an event that will happen in the next month on a particular day and then to describe it in as much detail as possible–who you are with, what you do and feel, and how and where it happens (“Imagine an event that will occur next month on a particular day. Please describe it with as many details as possible including who you were with, what you were doing and feeling, and how and when it occurred”/“Imagina un evento que va a pasar en el mes que viene en un dia particular. Por favor, describalo con lo mas detalles que es posible, con quien anda, que haces y sientes, y como y donde lo occure”). Additionally, the participant was then asked to imagine the impact the event would have on the whole community (collective future thought). Responses were scored on a scale of 1–5 for 7 characteristics of the description: amount of visual detail, clarity of the location, clarity of time of day, depth of emotion associated with the event, clarity of emotion associated with the event. This was scored separately for EFT and for collective EFT.

### Procedure

Testing was carried out in one session. Testing took place in dedicated testing rooms at Trinity College. All testing was done in Spanish by native Spanish speaking researchers. The total testing time ranged from 45 to 90 min. Questionnaires were administered after informed consent was obtained. The order of the memory tests was counter- balanced. Breaks were given if the participant complained of fatigue.

### Statistical analysis

To test the first hypothesis, to analyze reliability, the Cronbach α was determined in the case of internal consistency. The relationship between the cognitive measures and measures of acculturation and language (preferred language, years speaking a language, and years of education in a language) were measured using Pearson product moment correlations and univariate analysis of variance (ANOVA). Although the MIST variables were non-normally distributed (i.e., negatively skewed) as determined by a Shapiro–Wilk *W*-test (*p*s < 0.01), the results of the primary analysis did not change when a non-parametric approach to testing the statistical interaction was used. Cohen’s *d* was used and interpreted as small effect (0.2), medium (0.5), and large (0.8). All presented analyses were performed using IBM SPSS Statistics software (Version 22.0, SPSS Inc., Chicago, IL, USA).

## Results

### Characteristics of the participant sample

The demographics of the participants and cultural background variables are presented in Table 1. As can be seen, the participants were twice as many women as men, most were born in the U.S., and most spoke Spanish as their first language. On average they had a high school level of education.

### Psychometric properties of the MIST

Cronbach’s alpha is 0.91 for the individual scales (time, event, 2 min, 15 min, action, and verbal) and 0.70 for the eight individual trials, which is comparable to the English language version. Individual intercorrelations of the eight trials were significant at the *p* < 0.01 level. While performance on the MIST, shown in Tables 2, 3, is in the range of that for English speaking participants on the English language MIST, it is slightly lower.

**TABLE 2:** Spanish MIST performance *n* = 48.

	Mean (s.d.)	Range
2-min delay	6.98 (1.62)	2–8
15-min delay	5.42 (2.17)	0–8
Event-based cue	6.67 (1.81)	0–8
Time-based cue	5.85 (1.54)	2–8
Verbal response	6.56 (1.54)	2–8
Action response	5.97 (1.89)	0–8
Recognition	7.60 (0.64)	6–8
24 h recall	0.71 (0.89)	0–2
Total score	37.45 (8.85)	6–48
PM error score	1.42 (1.46)	0–8

**TABLE 3:** Acculturation and language variables and Memory for Intentions (MIST) variables correlations *r* and *p*-values *n* = 50.

	MIST time	MIST total	MIST PM error	MIST recognition
Years school	0.06 *p* = 0.69	0.12 *p* = 0.41	−0.07 *p* = 0.62	0.35* *p* = 0.01
Years speaking English	0.15 *p* = 0.30	0.19 *p* = 0.19	−0.17 *p* = 0.24	0.33* *p* = 0.02
Years speaking Spanish	−29* *p* = 0.04	−0.32* *p* = 0.03	0.24 *p* = 0.10	−0.22 *p* = 0.14
Years Education in U.S.	0.03 *p* = 0.85	0.10 *p* = 0.50	−0.01 *p* = 0.93	0.27 *p* = 0.06
Years of Education in Spanish	−0.02 *p* = 0.89	0.01 *p* = 0.96	0.04 *p* = 0.79	0.03 *p* = 0.82
Years of Education in English	0.04 *p* = 0.81	0.16 *p* = 0.29	−0.07 *p* = 0.63	0.29* *p* = 0.04
AMAS U.S.	0.29* *p* = 0.04*	0.32* *p* = 0.03	−0.10 *p* = 0.47	0.15 *p* = 0.29
AMAS C.O.	−0.17 *p* = 0.24	−0.20 *p* = 0.18	0.12 *p* = 0.41	−0.21 *p* = 0.15

AMAS U.S., Abbreviated Multidimensional Acculturation Scale, United States culture; AMAS C.O., Abbreviated Multidimensional Acculturation Scale, country of origin culture.

*p < 0.05.

### Acculturation, language, and cognitive measures

#### Prospective memory

##### MIST

The Pearson product moment correlations for the MIST and measures of acculturation, language, and education are provided in Table 3. As can be seen, the MIST Recognition item was significantly positively correlated with years of education, years of speaking English, and years of education in English. Years of speaking Spanish was negatively correlated with MIST 15-min cues and time-based cues. Acculturation into U.S. culture was significantly positively correlated with time-based cues and MIST total score.

One-way analysis of variance (ANOVA) was computed for preferred language (English, Spanish, and both). There were no differences for the MIST scores (total score, recognition score, and total PM errors).

In terms of other demographic variables (gender, age), a point by serial correlation revealed no significant relationship between gender and performance on the MIST variables. For age, not surprisingly, a Pearson’s product moment correlation was significant indicating lower performance associated with older age (MIST total score *r* = −0.35; *p* < 0.05).

Data for the CAPM are presented in Table 4.

**TABLE 4:** Spanish CAPM performance *n* = 48.

	Mean (s.d.)	Range
BADL	1.56 (0.54)	1–4
IADL	2.03 (0.56)	1–3.70

#### Working memory

The measure of working memory, the Digit Span Forward and Backward, raw score was a mean of 7.48 (s.d. 1.45) and a range of 4–10 forward and 7.36 (s.d. 2.15) with a range of 4–11 backward.

#### Autobiographical memory

Table 5 presents the overall performance on the three scales of the TALE.

**TABLE 5:** Spanish TALE performance *n* = 48.

	Mean (s.d.)	Range
Self-continuity	15.29 (4.6)	5–25
Social bonding	17.29 (4.32)	6–25
Directing Behavior	18.46 (3.83)	7–25

#### Future thought

Overall scores for episodic future thought was a mean of 6.48 (± 3.60) and for collective future thought was a mean of 3.00 (± 2.14).

#### Relationship between PM and other cognitive measures

Pearson product moment correlations revealed no significant relationship between the MIST (total score, total PM errors) and the CAPM (IADL, BADL) or the TALE (self-continuity, social bonding, and directing). The MIST (total score) was significantly related to the test of episodic future thought (*r* = 0.32, *p* < 0.01).

## Discussion

The current study is the first, to our knowledge, to investigate the measurement of PM in healthy Spanish speakers and the first to examine sociocultural factors in PM. The first aim of the present study was to determine the psychometric properties of the Spanish MIST and determine some baseline normative data. In terms of psychometric properties, the Spanish language MIST demonstrates adequate reliability, and the normative data suggest that performance is comparable to that found with English language speakers on the English language MIST, including reduced performance with increasing age (Raskin et al., 2010).

The second aim was to determine the effects of acculturation on measures of PM, working memory, autobiographical memory, and episodic future thought in a Spanish speaking population living in the U.S. and the interrelationship between PM and the other measures. There were few effects found of acculturation on the working memory task or the autobiographical memory test.

For PM, using the MIST, there were significant positive effects of education, and years of speaking English, and years of education in English on the recognition item. There were also significant effects of years of speaking Spanish such that years of speaking Spanish was negatively correlated with both the time-based items and the total MIST score. This suggests that the current Spanish version of the MIST may be impacted by education and language factors. Future studies should examine the individual items and, perhaps, design new test items that are less influenced by these factors. Although the MIST was translated and back translated by bilinguals researchers, previous authors have pointed out that this may not be adequate and so individual item translation should be reexamined (Brickman et al., 2006).

The effects of education and language spoken were most pronounced for recognition as compared to recall of PM items. We are not aware of previous students that have looked at PM recognition memory specificity in Spanish speakers. However, previous studies have suggested a similar effect whereby North American participants showed better performance in memory specificity compared to East Asians on measures of recognition memory (Leger and Gutchess, 2021). These authors speculated several possible explanations including the possibility that North Americans store more detailed representations of previously studied items. Future studies should also explore fluency in English versus Spanish, as this can also impact recognition performance (Francis and Gutiérrez, 2012).

It is interesting that there were language effects on the objective measure of prospective memory but not on the self-report measure. This suggests that language and educational effects are specific to the types of questions being asked in the MIST. It is possible that there are specific items that are presented in the MIST that are used less frequently in other cultures. It is, however, not surprising to find differences between these two measures as performance on them has been shown previously to be poorly related (Raskin et al., 2018).

Total score on the MIST was also related to acculturation as measured by the AMAS. This is consistent with the findings of Tureson et al. (2021) using the English language version of the MIST in a Latinx populations. A similar finding was reported when comparing European American to Chinese participants such that the European American participants reported greater detail when asked to imagine future events (Wang et al., 2011). This was suggested to provide support for the constructive-episodic-simulation hypothesis (Addis and Schacter, 2008). This warrants further study in individuals from Spanish speaking countries.

This study has several important limitations, the first being a sample size that was too small to carry out further analyses on other demographic variables such as measures of bilingualism and preferred language. Second, our participants had cultural ties to several different Latin American regions with unique cultures and vernaculars. In addition, several of our measures have not been previously validated.

Overall, these data lend support to the growing recognition of a need to be aware of issues of language and culture in neuropsychological assessment (e.g., Rivera Mindt et al., 2008; Judd et al., 2009), and obtaining adequate and specific normative data (e.g., Rivera et al., 2019; Morlett Paredes et al., 2021). However, they also suggest areas where further research might help explain specific differences in memory processing that are related to language and culture, including different cultural uses of memory (e.g., Alea et al., 2019).

## Data availability statement

The raw data supporting the conclusions of this article will be made available by the authors, without undue reservation.

## Ethics statement

The studies involving human participants were reviewed and approved by the Trinity College Institutional Review Board. The patients/participants provided their written informed consent to participate in this study.

## Author contributions

All authors made substantial contributions to the conception of the study, assisted in data collection and data analysis, and participated in the writing of the manuscript.
